# A Systematic Review of Clinical Trials Using mRNA Vaccines for Infectious Diseases other than COVID-19

**DOI:** 10.3389/bjbs.2025.14557

**Published:** 2025-07-18

**Authors:** Athina Sofroniou, Anna Ridley

**Affiliations:** Faculty of Medicine and Dentistry, Queen Mary University of London, London, United Kingdom

**Keywords:** mRNA, safety, clinical trials, infectious diseases, vaccines

## Abstract

**Background:**

Although mRNA-based vaccines have been in development for over two decades, their widespread use only emerged during the COVID-19 pandemic. The success of these vaccines has brought mRNA technology to the forefront of efforts to develop novel vaccines. However, as this is a rapidly evolving field, there is a need for a comprehensive and up-to-date overview of the current evidence base to guide further research and development. This study, therefore, systematically reviewed the literature on clinical trials using mRNA vaccines for infectious diseases other than COVID-19.

**Methods:**

A systematic review of the literature, following the PRISMA 2020 guidelines, identified clinical trials in infectious diseases other than COVID-19. PubMed and ClinicalTrials.gov were screened for such clinical trials using search terms related to mRNA vaccines, and the results of the two independent searches were combined. Clinical trials using mRNA vaccines against either COVID-19 or non-communicable diseases were removed, as were duplicated studies. The remaining clinical trials were then stratified based on pathogen, status, and phase.

**Results:**

Nine hundred and seventy-six clinical trials were identified, of which 83 met the inclusion criteria. These included candidate mRNA vaccines against 14 viral, two bacterial and one protozoan infection. Of these, 43 trials have concluded, 21 are active, and a further 12 are recruiting, with the remaining not yet recruiting, enrolling by invitation, or withdrawn. Of the 43 completed clinical trials, 26 were phase I trials, eight were phase I/II trials, three were phase II trials, and six were phase III trials. The clinical trials captured in this systematic review included combined vaccines, with two or more vaccines administered at the same time, and mRNA vaccines designed to encode pathogen structural components, in addition to pathogen-specific antibodies.

**Conclusion:**

This systematic review identified clinical trials investigating mRNA vaccine candidates against multiple infectious diseases, other than COVID-19, with the majority targeting viral infections. Despite the lack of long-term data, this systematic review suggests that these mRNA vaccine candidates are safe and effective with the potential to shape the field of preventive medicine. Beyond the prevention of infectious diseases, mRNA vaccines are showing promise against cancer and potential applications in autoimmune and other diseases.

## Introduction

Vaccination is widely regarded as one of modern medicine’s most successful and cost-effective prophylactic interventions, leading to the successful eradication of smallpox in 1980 and limiting the prevalence and incidence of many other infectious diseases [1]. Despite several advancements and breakthroughs in conventional vaccine development, there are still no vaccines available for many infectious diseases [2]. Globalisation and climate change have accelerated the diffusion of infectious pathogens through human and animal vectors, increasing the risk of emerging and re-emerging pathogens spreading [3]. This highlights the need for versatile vaccine platforms with easily scalable production for rapid response to such threats. One promising technology lies in messenger ribonucleic acid (mRNA) vaccines, which have the potential to revolutionise the field of preventive medicine by overcoming the hindrances presented by conventional vaccination methods.

Traditional vaccination methods have proved ineffective against pathogenic microorganisms with more complex virulence mechanisms and immune evasion strategies, such as the human immunodeficiency virus (HIV) and the malaria parasite *Plasmodium falciparum* [4]. Unlike live-attenuated vaccines, mRNA vaccines do not contain live or replicating pathogens and therefore carry no risk of reversion to the wild type and causing infection. Since mRNA remains in the cytoplasm and does not integrate into the host genome, the potential for insertional mutagenesis that can be theoretically associated with some DNA-based or viral vector vaccines is avoided [5]. Although there is no evidence of vaccines against infectious disease leading to such mutagenesis, the use of integrative vectors, such as those used for gene therapy in severe combined immunodeficiency, has been associated with insertional oncogenesis [6]. Given public concerns around vaccination and the increase in vaccine hesitancy clear information regarding vaccines is essential.

Currently, mRNA vaccine constructs exist in two primary forms: conventional/non-replicating mRNA (NRM) and self-amplifying mRNA (SAM) [7]. Essentially, both types of mRNA vaccines consist of a modified mRNA molecule and a delivery system that encapsulates the mRNA payload [8, 9] but differ in design and mode of action. Specifically, SAM vaccines incorporate genetic information that encodes the open reading frame (ORF) of the target antigen, a 5′ 7-methylguanosine triphosphate (m7G) cap structure, 5′ and 3′ untranslated regions (UTRs), a 3′-poly A tail, and a viral replicase complex that allows for mRNA self-amplification within cells [8–10]. In contrast, NRM vaccines have the typical structure of a eukaryotic mRNA [11] and thus lack the viral replication machinery that characterises SAM vaccines, and do not undergo any amplification steps following endosomal escape [8, 10]. This lack of direct antigen expression in NMR vaccines eliminates the risk of anti-vector immunity [12], but the viral replication machinery of SAM allows for an improved, prolonged expression of antigens and subsequent immune recognition, ultimately increasing the production of pro-inflammatory cytokines and immune cell activation [12]. SAM vaccines show promise in stimulating strong immunological responses, but prolonged activation of the innate immune response can cause downstream effects in mRNA expression, leading to a decline in mRNA vaccine efficacy [13].

To date, multiple mRNA vaccines designated against challenging pathogens, such as HIV, the rabies virus, the respiratory syncytial virus (RSV), the Zika virus, the cytomegalovirus (CMV), and the influenza virus [14–16], among other pathogenic microorganisms, have made it into clinical trials. However, as this is a relatively new approach in a rapidly evolving field, there is a need for a comprehensive, up-to-date overview of current clinical trials to guide further research and development. Although literature reviews of mRNA vaccines exist [4, 15, 17], along with systematic reviews of mRNA vaccine against COVID-19 [18,19], to our knowledge this is the first systematic review of mRNA vaccines for infectious diseases other than COVID-19. Given the increase in mRNA vaccine trials, the shift in vaccine platforms, and the emergence of new infectious disease threats, providing a comprehensive overview of ongoing and completed clinical trials of mRNA vaccines across multiple pathologies is critical to guide further research and development.

## Methodology

A systematic review of the existing literature was undertaken, following the Preferred Reporting Items for Systematic Review and Meta-Analysis (PRISMA) 2020 guidelines to evaluate the current state of clinical trials in mRNA vaccines for infectious diseases other than COVID-19. Keyword searches were conducted in the PubMed database, to identify relevant, peer-reviewed articles published. The search strategy consisted of the following combination of keywords: [(infection) AND (mRNA) AND (clinical trial) NOT (COVID)]. Additionally, the ClinicalTrials.gov online database was searched using the keyword “mRNA vaccines” to identify individual clinical trials using mRNA vaccines for infectious diseases other than COVID-19. The title and abstract of each article generated by the keyword searches were screened to identify eligible studies and exclude irrelevant studies by two independent reviewers, and any ambiguities were clarified through discussion.

**FIGURE 1:**
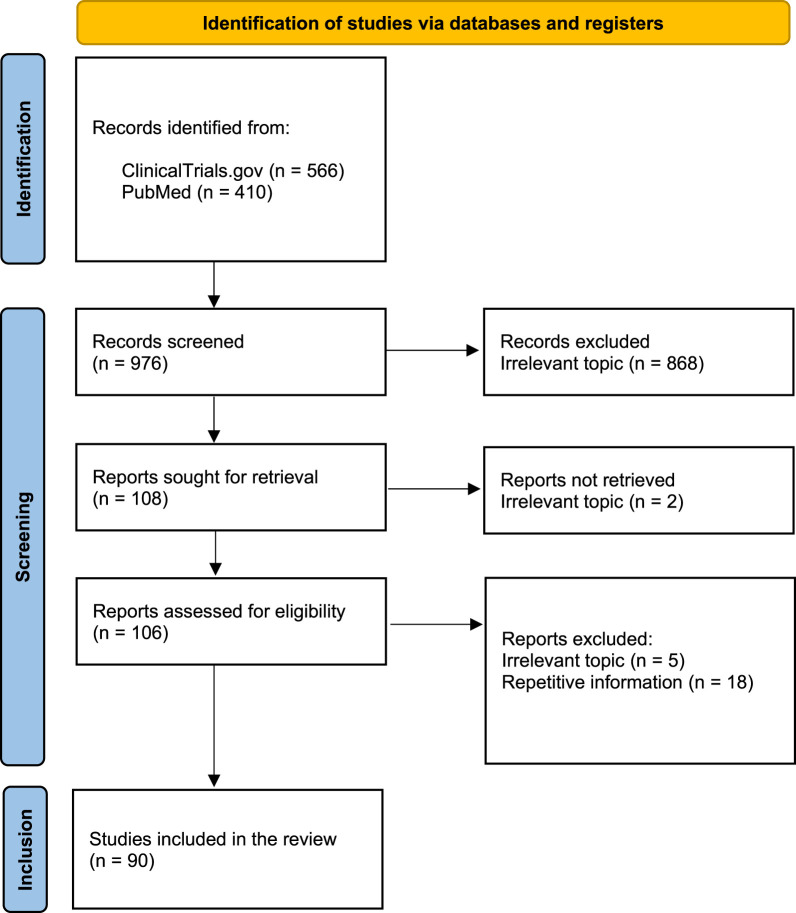
PRISMA flow diagram showing the process of study selection for records retrieved from the ClinicalTrial.gov and PubMed databases.

Following the initial evaluation, the full texts of the remaining search outputs were assessed for inclusion based on the following eligibility criteria: i) clinical trials in humans ii) the study reports on mRNA vaccines for infectious diseases other than COVID-19, iii) the article is written in English. Irrelevant studies, such as those articles focusing on mRNA vaccines for the treatment of cancer or COVID-19, along with duplicates were excluded to maintain a focus on mRNA vaccines against infectious diseases other than COVID-19. Details of the study selection process are illustrated in the PRISMA flow diagram (Figure 1), together with the justifications for their elimination.

## Results

A total of 976 records were identified in the initial search. At screening, 868 were classified as not eligible due to their focus on topics irrelevant to the scope of this study and were removed. The title, diseases, and intervention details of 108 records were independently screened for relevance by two individuals. A total of 106 full-text records were then assessed for eligibility based on the established eligibility criteria, and any disagreements were resolved through discussion between the two authors. Of these 106 records, five studies were removed as the study design fell outside the scope of mRNA vaccines. An additional 18 entries were excluded due to duplication, as they consisted of repetitive information. Finally, 83 studies were included in this review. Of these, 82 were clinical trials sourced from the ClinicalTrials.gov database and one study was sourced from the PubMed database. A summary of the study characteristics of all completed clinical trials using mRNA vaccines against infectious diseases identified is presented in Table 1 and the remaining clinical trials, those that are ongoing, are presented in Table 2.

**TABLE 1:** Characteristics of completed clinical trials using mRNA vaccine candidates against infectious diseases other than COVID-19.

Pathogen group	Condition(s)	Intervention(s)	Immunogen(s)	Route of administration	Sponsor	Phase	NCT Number	Ref
Virus	CHIKV	mRNA-1944	CHKV-24	IV	ModernaTX, Inc.	I	NCT03829384	[20]
		mRNA-1388	CHIKV West African strain 37,997 full structural polyprotein	IM		I	NCT03325075	[21]
	CMV	mRNA-1647, mRNA-1443	NS	NS	ModernaTX, Inc.	I	NCT03382405	[22]
		mRNA-1647	gB	IM	ModernaTX, Inc.	II	NCT04232280	[23]
						I	NCT05105048	[24]
	HIV	iHIVARNA-01	NS	InG	Judit Pich Martínez	I	NCT02413645	[25]
	Influenza	VAL-506440	HA	IM	ModernaTX, Inc.	I	NCT03076385	[26]
		VAL-339851	HA	IM	ModernaTX, Inc.	I	NCT03345043	[27]
		mRNA-1010	HA	IM	ModernaTX, Inc.	III	NCT05827978	[28]
						III	NCT05566639	[29]
						III	NCT05415462	[30]
						I/II	NCT04956575	[31]
						II	NCT05868382	[32]
		mRNA-1020, mRNA-1030	HA, NA	IM	ModernaTX, Inc.	I/II	NCT05333289	[33]
		mIRV, bIRV, qIRV	HA	IM	Pfizer	I/II	NCT05052697	[34]
		mRNA-1011, mRNA-1012	HA	IM	ModernaTX, Inc.	I/II	NCT05827068	[35]
		MRT5407	HA	IM	Sanofi Pasteur	I/II	NCT05553301	[36]
		MRT5410	HA	IM	Sanofi Pasteur	I/II	NCT05624606	[37]
		MRT5413	HA	IM	Sanofi Pasteur	I/II	NCT05650554	[38]
		CVSQIV	HA	IM	CureVac	I	NCT05252338	[39]
		Monovalent mRNA	HA	IM	Sanofi Pasteur	I	NCT06118151	[40]
		GSK4382276A	NS	IM	GlaxoSmithKline	I	NCT05446740	[41]
		sa-mRNA (Dose 1–3)	HA	IM	Seqirus	I	NCT06028347	[42]
		H3 mRNA/LNP	HA	IM	Sanofi Pasteur	I	NCT05829356	[43]
		Flu mRNA	NS	IM	GlaxoSmithKline	I/II	NCT05823974	[44]
		Quadrivalent influenza modRNA	HA of 4 seasonal strains	IM	Pfizer	III	NCT05540522	[45]
		Monovalent mRNA NA	NA	IM	Sanofi Pasteur	I	NCT05426174	[46]
		PF-07852352, PF-07836391, PF-07836394, PF-07836395, PF-07836396, PF-07867246, PF-07871987, PF-07914705, PF-07915048/ Influenza saRNA	NS	IM	Pfizer	I	NCT05227001	[47]
	NiV	mRNA-1215	Prefusion stabilised F covalently linked to G monomer (Pre-F/G) of Malaysian strain NiV	IM	NIAID	I	NCT05398796	[48]
	RABV	CV7201	RabG	NS	CureVac	I	NCT02241135	[49]
		CV7202	NS	IM	CureVac	I	NCT03713086	[50]
	RSV	mRNA-1345	RSV preF	IM	ModernaTX, Inc.	I	NCT04528719	[51]
						III	NCT06060457	[52]
						III	NCT05330975	[53]
		mRNA-1777	RSV preF	IM	Merck Sharp & Dohme Corp.	I	N/A	[54]
	ZIKV	mRNA-1325	NS	NS	ModernaTX, Inc.	I	NCT03014089	[55]
		mRNA-1893	NS	NS	ModernaTX, Inc.	I	NCT04064905	[56]
						II	NCT04917861	[57]
	HMPV and HPI	mRNA-1653	anti-hMPV, anti-PIV3 (NS)	IM	ModernaTX, Inc.	I	NCT04144348	[58]
				NS		I	NCT03392389	[59]
	RSV and HMPV	RSV/hMPV mRNA LNP 1–2, RSV mRNA LNP, hMPV mRNA LNP	RSV: preF, HMPV: preF	IM	Sanofi Pasteur	I	NCT06237296	[60]
	RSV and Influenza	RSV: RSVpreF and Influenza: gIRV	RSV: preF, Influenza: HA (H1N1 A/Sydney, H3N2 A/Darwin, B/Austria, B/Phuket)	IM	Pfizer	I	NCT05788237	[61]
Protozoa	Malaria	BNT165b1	PfCSP	IM	BioNTech SE	I	NCT05581641	[62]

Abbreviatons: CHIKV: chikungunya virus, CMV: cytomegalovirus, gB: Glycoprotein B, HA: hemagglutinin, HIV: human immunodeficiency virus, HMPV: human metapneumovirus, HPI: human parainfluenza infection, InG: Inguinal Intranodal, IM: intramuscular, IV: intravenous, NA: neuraminidase, NiV: nipah virus, NS: not specified, RABV: rabies virus, Ref: Reference, RSV: respiratory syncytial virus, ZIKV: Zika Virus.

**TABLE 2:** Characteristics of ongoing clinical trials using mRNA vaccine candidates against infectious diseases other than COVID-19.

Pathogen group	Condition(s)	Intervention(s)	Immunogen(s)	Route of administration	Sponsor	Phase	Status	NCT Number	Reference
Virus	CMV	mRNA-1647	gB	IM	ModernaTX, Inc.	I	Recruiting	NCT05683457	[63]
	EBV	mRNA-1189	NS	IM	ModernaTX, Inc.	I		NCT05164094	[64]
	HSV-2	BNT163	gC2, gD2, gE2	IM	BioNTech SE	I		NCT05432583	[65]
	HIV	CH505M5 N197D mRNA-gp160, CH505 TF mRNA-gp160	HIV-1 envelope protein gp160	IM	NIAID	I		NCT06557785	[66]
	Influenza	mRNA-1010	HA	IM	ModernaTX, Inc.	II		NCT05606965	[67]
		DCVC H1 HA mRNA-LNP	H1 HA	IM	NIAID	I		NCT05945485	[68]
		Flu Pandemic mRNA (Dose level 1–7)	HA	IM	GlaxoSmithKline	I/II		NCT06382311	[69]
	RSV	mRNA-1345	RSV preF	IM	ModernaTX, Inc.	II		NCT06143046	[70]
		IN006	RSV preF	IM	ModernaTX, Inc.	I		NCT06645665	[71]
		Investigational RSV vaccine 1–6	NS	IM	GlaxoSmithKline	I		NCT06573281	[72]
		JCXH-108	RSV preF	IM	Immorna Biotherapeutics, Inc.	I		NCT06564194	[73]
	RSV and HMPV	mRNA-LNP hMPV/RSV, monovalent hMPV, monovalent rsv	RSV: preF, HMPV: preF	IM	Sanofi Pasteur	I/II		NCT06686654	[74]
	CMV	mRNA-1647	gB	IM	ModernaTX, Inc.	II	Enrolling by invitation	NCT04975893	[75]
	CMV	mRNA-1647	gB	IM	ModernaTX, Inc.	III	Active, not recruiting	NCT05085366	[76]
	EBV	mRNA-1195, mRNA-1189	NS	IM	ModernaTX, Inc.	II		NCT05831111	[77]
	VZV	PF-07915234, PF-07915234, PF-07921188, PF-07921186, PF-07921188	gE	IM	Pfizer	II		NCT05703607	[78]
		IN001	gE	IM	Shenzhen Shenxin Biotechnology Co., Ltd	I		NCT06375512	[79]
		mRNA-1468	gE	IM	Pfizer	I/II		NCT05701800	[80]
	HIV	mRNA-1644	eOD-GT8 60 mer	IP	International AIDS Vaccine Initiative	I		NCT05414786	[81]
		mRNA-1644, mRNA-1644v2-Core	eOD-GT8 60mer, Core-g28v2 60 mer	IM	International AIDS Vaccine Initiative	I		NCT05001373	[82]
		BG505 MD39.3 mRNA, BG505 MD39.3 gp151 mRNA, BG505 MD39.3 gp151 CD4KO mRNA	HIV-1 BG505 envelope protein gp151	IM	NIAID	I		NCT05217641	[83]
	Influenza	MRT5421, MRT5434, MRT5429	HA	IM	Sanofi Pasteur	I/II		NCT06361875	[84]
		GSK4382276A	NS	IM	GlaxoSmithKline	II		NCT06431607	[85]
		ARCT-2138	HA, NA	IM	Arcturus Therapeutics, Inc.	I		NCT06125691	[86]
		H1ssF_3928	H1-specific	IM	NIAID	I		NCT05755620	[87]
	RSV	mRNA-1345	RSV preF	IM	ModernaTX, Inc.	II		NCT06097299	[88]
				IM		III		NCT06067230	[89]
				IM		II/II		NCT05127434	[90]
		RSVPreF3 OA		IM	GlaxoSmithKline	III		NCT06374394	[91]
		RSV mRNA-LNP (Formulation 1–2)		IM	Sanofi Pasteur	I/II		NCT05639894	[92]
	RSV and HMPV	mRNA-1345, mRNA-1365	RSV: preF, HMPV: preF	IM	ModernaTX, Inc.	I		NCT05743881	[93]
Bacteria	*Borrelia burgdorferi*:	mRNA-1975, mRNA-1982	OspA	IM	ModernaTX, Inc.	I/II		NCT05975099	[94]
	TB	BNT164a1, BNT164b1	NS	IM	BioNTech SE	I/II		NCT05547464	[95]
		BNT164a1, BNT164b2	NS	IM	BioNTech SE	I		NCT05537038	[96]
Virus	HIV	mRNA-1645-eODGT8, mRNA-1645-CoreG28v2, mRNA-1645-N332GT5	eOD-GT8 60mer, Core-g28v2 60 mer, N332-GT5 gp151	IM	HIV Vaccine Trials Network	I	Not yet recruiting	NCT06694753	[97]
	Influenza	ARCT-2304	HA	IM	Arcturus Therapeutics, Inc.	I		NCT06602531	[98]
	JEV	GBP560 A,B	NS	IM	SK Bioscience Co., Ltd.	I/II		NCT06680128	[99]
	RSV	IN006	RSV preF	NS	Shenzhen Shenxin Biotechnology Co., Ltd	I		NCT06287450	[100]
		STR-V003	NS	IM	Starna Therapeutics	I/II		NCT06344975	[101]
	CMV	mRNA-1647	gB	IM	ModernaTX, Inc.	II	Withdrawn	NCT06133010	[102]

Abbreviatons: CMV: cytomegalovirus, EBV: Epstein-Barr Virus, gB: Glycoprotein B, gC2: Glycoprotein C2, gD2: Glycoprotein D2, gE: Glycoprotein E, gE2: Glycoprotein E2, HA: hemagglutinin, HIV: human immunodeficiency virus, HMPV: human metapneumovirus, HSV-2: Herpes Simplex Type 2, IM: intramuscular, IP: intraperitoneal, JEV: japanese encephalitis virus, NA: neuraminidase, NS: not specified, Ref: Reference, RSV: respiratory syncytial virus, TB: tuberculosis, VZV: varicella zoster virus.

The purpose of this review was to provide a comprehensive overview of clinical trials using mRNA vaccines for infectious diseases other than COVID-19. mRNA technology has emerged as a promising vaccine platform with revolutionary potential in preventive medicine. A significant proportion of the literature is centred on mRNA vaccines against COVID-19, but substantial research has also been conducted on mRNA vaccines for other infectious diseases. Of the clinical trials included in this review 43 trials were reported as completed. Of the 40 ongoing clinical trials 21 are active, 12 are recruiting, five are not yet recruiting, one is enrolling by invitation only, and one has been withdrawn. Of the 43 completed clinical trials 26 were phase I trials, eight were phase I/II trials, three were phase II trials and six were phase III trials. These included candidate mRNA vaccines against 14 viral infections, two bacterial infections and one protozoan infection.

### Completed Clinical Trials for mRNA Vaccine Candidates Against Viral Infections

Viral infections are an obvious target for mRNA vaccines due to the rapid rate of mutation seen in many viruses and their use of specific surface proteins for infection. Over 60 candidate mRNA vaccines were elucidated by this systematic review, against 14 different viral pathogens with the results of the completed clinical trials summarised below.

### Chikungunya

The safety and pharmacology of the mRNA vaccine candidate mRNA-1944 were evaluated in a phase I clinical trial (NCT03829384) [20]. The vaccine, encoding the heavy and light chains of a CHIKV-specific monoclonal neutralising antibody, CHKV-24, demonstrated neutralising activity against CHIKV in humans at titres considered therapeutically optimal and remained durable for at least 4 months at dosages of 0.3 and 0.6 mg kg^−1^ with no indication of severe adverse events [103]. A second dose of 0.3 mg kg^−1^ stimulated a 1.8-fold increase in neutralising antibody titres in serum and durability, although neutralising antibody responses were only assessed for up to 48 h following intravenous infusion; therefore, further evaluation in the form of extended studies is necessary to determine the efficacy of mRNA-1944 in the long term.

Taking a different approach Moderna TX, Inc. has developed an mRNA-LNP CHIKV vaccine construct encoding the full structural polyprotein of the CHIKV West African strain 37,997, mRNA-1388, which has completed a phase I clinical trial (NCT03325075) [21]. The results demonstrated favourable tolerability at various dosages (i.e., 25, 50, and 100 μg) with mild solicited local (e.g., pain and tenderness) and systemic (e.g., headache, generalised myalgia, and fatigue) adverse reactions. Neutralising antibody and CHIKV binding antibody titres increased in a dose-dependent manner in all participants, and seroconversion was higher in the 100 μg dosage group. Although mRNA-1388 produced strong and durable neutralising antibody responses, the study was conducted in a non-endemic area. Additionally, the impact of sex and ethnicity on immune responses was not assessed; thus, there is a need for further research on the at-risk populations. Since the vaccine immunogen was derived from a non-predominant global genotype, further investigation would also be necessary to determine whether any antigenic variations across strains will affect the neutralising activity of mRNA-1388 sera.

### Cytomegalovirus

Bivalent CMV mRNA-LNP vaccine formulations, mRNA-1647 and mRNA-1443, which co-express both gB and PC glycoproteins have been explored in a phase I clinical trial (NCT03382405) [22]. Only mRNA-1647 moved forward to phase II (NCT04232280) [23], after the clinical material of mRNA-1443 failed to meet internal standards after 1 year of storage, raising concerns about its safety and efficacy due to poor long-term stability [104]. The safety, reactogenicity, and immunogenicity of mRNA-1647 were also explored in a further phase I clinical trial [24] with results showing the generation of neutralising antibodies and a sustained immune response 6 months after the third dose [105].

### Human Immunodeficiency Virus Type 1

A naked mRNA (iHIVARNA) therapeutic vaccine candidate expressing a novel HIV immunogen in conjunction with TriMix mRNAs was recently evaluated in chronic HIV-1-infected patients on antiretroviral therapy in a phase I clinical trial (NCT02413645) [25], where it demonstrated promising outcomes. TriMix mRNAs are a combination of three mRNA sequences that encode for activation molecules (Human CD4L, constitutively active TLR4 [(caTLR4]), and CD70), which enhance T-cell activation and subsequent antigen presentation by dendritic cells [106]. Three intranodal immunisations of iHIVARNA at varying doses (100–1200 μg) were well tolerated by the study participants, with the majority of adverse reactions being unrelated to the vaccine [107]. The vaccine demonstrated good safety with moderate HIV-1-specific T cell responses and has now progressed to phase II for further evaluation. Interestingly, the authors also reported an interaction between increasing viral load titres and immune responses that target vaccine-encoded epitopes.

### Influenza

In total 30 clinical trials using mRNA vaccines against influenza were captured in this systematic review, 21 of which have concluded. The earliest influenza mRNA-based vaccines to enter clinical trials, mRNA-H10N8 (NCT03076385) [26] and mRNA-H7N9 (NCT03345043) [27], were well tolerated with strong humoral immune responses in humans when administered intramuscularly as a two-dose regimen [108]. Interestingly, intradermal immunisation led to high rates of solicited adverse events, and enrolment was discontinued [108].

A further mRNA vaccine candidate, mRNA-1010, has been extensively studied across different clinical trials [28–30] assessing its safety, immunogenicity, and potential efficacy as a seasonal flu vaccine. The vaccine demonstrated a strong safety profile across different studies, similar to that of Fluarix, a licensed influenza vaccine comprising inactivated HA and NA viral particles) [29, 30]. mRNA-1010 also demonstrated favourable immunogenicity against influenza A strains, H1N1 and H3N2, with higher rates of participants achieving protective antibody titres when compared to Fluarix [29, 30]. However, no statistical difference was noted in disease prevention between mRNA-1010 and Fluarix [29], with mRNA-1010 demonstrating lower efficacy against influenza B strains, Victoria- and Yamagata-lineage, and slightly higher influenza-like-illness in older adult populations [30].

Optimal dosing for mRNA-1010 was explored in clinical trials (NCT04956575) [31] and (NCT05868382) [32]. Safety and tolerability were best balanced in the 25 μg dose group [42], while a different formulation of mRNA-1010, mRNA-1010.6 (medium dose), showed an optimal balance between immunogenicity and tolerability [32].

A robust immune response with a favourable safety profile, despite some increased reactogenicity, was also demonstrated by mRNA-1020 in a phase I/II clinical trial (NCT05333289) [33], in which both mRNA-1020 (Dose Level A/B) and −1030 (Dose Level B) outperformed mRNA-1010.

In a further phase I/II clinical trial (NCT05052697) [34], the safety, tolerability, and immunogenicity of several modified mRNA vaccine candidates were evaluated in younger and older adults. The results suggest that the quadrivalent vaccine (qIRV + QIV) generated the strongest immune responses, especially against influenza A strains. An immune response to all strains was also observed in groups that received the bivalent formulation (bIRV AB). Immunogenicity was higher in younger adults, although older adults who received the qIRV or bIRV formulations showed improved immune responses. The modified mRNA vaccines were also well tolerated with no major safety concerns, making them promising mRNA vaccine candidates for vaccination in broad age groups.

### Nipah Virus

The Nipah virus (NiV) is a highly pathogenic zoonotic virus belonging to the Henipavirus genus that can lead to fatal neurological and respiratory diseases in both humans and animals. Despite the lack of licensed NiV vaccines, research and development efforts are underway, with some vaccine candidates showing promise. A phase I clinical trial is the first study of mRNA-1215 in healthy adults to evaluate the safety, tolerability, and immunogenicity of the NiV mRNA vaccine candidate mRNA-1215 (NCT05398796) [48]. The study was completed in September 2024, but the results are yet to be made available.

### Rabies

The safety, reactogenicity, and immunogenic potential of the RABV mRNA-LNP vaccine candidate CV7201, which encodes the rabies virus glycoprotein (NCT02241135) [49] were evaluated in a phase I clinical trial. CV7201 was generally well tolerated, but the ability to elicit adequate immune responses was heavily contingent on the route of administration, requiring intradermal or intramuscular delivery employing specialised devices to attain the desired immune response [109]. Because of this, further development of this vaccine candidate was put on hold in favour of a different formulation, CV7202, which encompasses the same vaccine immunogen as CV7201 and has been proven to induce immunological responses in non-human primates, comparable to those elicited by commercially available vaccines in a phase I clinical trial, (NCT03713086) [50].

Intramuscular immunisation with CV7202 was generally well tolerated by the study participants, with no immediate adverse events and no vaccine-related serious adverse events or adverse events of particular concern throughout the trial [50]. However, a high rate of early-onset reactogenicity involving solicited local and mild-to-moderate systemic adverse events was observed in participants receiving the 5 μg regimen. Injection site pain was experienced by the majority of subjects, with one instance being classified as severe, while the majority of the systemic adverse events included headache, fatigue, myalgia, and chills [50]. When administered at 1 or 2 μg, CV7202 demonstrated a more favourable reactogenicity profile than CV7201 [50]. A two-dose regimen of the CV7202 vaccine at both 1 and 2 μg doses was also able to induce strong and durable neutralising antibody responses [50]. This indication was particularly significant as it suggests that CV7202 could be a promising candidate for further development, considering its low reactogenicity and the generation of robust neutralising antibody responses at both 1 and 2 μg doses: this makes it an efficient and well-tolerated vaccine alternative. Moreover, a two-dose regimen of CV7202 at both 1 and 2 μg doses resulted in a significant increase in RABV-G-specific IgG antibody titres [50], further corroborating the potential of this vaccine candidate.

### Respiratory Syncytial Virus

Several potentially promising mRNA-based vaccine candidates against RSV have been investigated in clinical trials. mRNA-1777 encodes the F protein of RSV stabilised in the prefusion conformation. The F protein exists in two forms; a metastable prefusion conformation and a post-fusion conformation [54]. Studies have shown that human antibodies specifically target the prefusion conformation of the F protein upon natural exposure to RSV [54], making this prefusion cofirmation an important vaccine target antigen for immune recognition. A single intramuscular injection of mRNA-1777 was generally well tolerated by participants in all dosage groups, with no serious vaccine-related adverse events reported [54]. The vaccine also successfully induced RSV prefusion F-specific neutralising antibody responses against both RSV subtypes in young adults and older participants, targeting key functional sites of the F protein. In this clinical trial participant immune responses were predominantly seen to be mediated by CD4^+^ T cells, where as natural infection results in a predominant CD8^+^ T cell response, indicating the potential for variations between vaccine-induced immune responses and natural infection.

A further vaccine, mRNA-1345, has been purposefully designed and codon-optimised to improve the translation efficacy and immunogenicity of an earlier vaccine candidate, mRNA-1777. The results from a phase I clinical trial in healthy adults and children (NCT04528719) [51] demonstrated strong and durable immune responses after a single injection, characterised by a robust increase in neutralising antibody titres and prefusion F binding antibodies [110]. In older adults, a fold-increase of ≥10.2 for RSVA and a ≥5.3-fold increase for RSVB was observed in neutralising antibody levels at month 1 post-vaccination, with persisting antibody titres above baseline levels for 12 months [110]. Even stronger immune responses were demonstrated in younger adults (RSVA: 20.0–23.5-fold increase, RSVB: 11.7-16.0-fold increase), with persisting antibody titres for 6 months [111]. The vaccine was also well tolerated by both older and younger populations, even after a booster dose [110, 111]. Reactions were generally mild to moderate in severity, with no major safety concerns [110, 111]. Reactogenicity was more pronounced in older adults following a booster dose at 12 months and comparable to the first injection in terms of intensity, onset, and persistence [51].

The ConquerRSV study, a global randomised, double-blind, placebo-controlled phase II/III clinical trial (NCT05127434) [90], in which 36,814 adults were enrolled, showed that mRNA-1345 can induce a significant increase in RSV-neutralising and preF-binding antibodies across diverse older adult subgroups, including high-risk populations. This supports previous findings [112]. As of 31 May 2024, mRNA-1345 (mRESVIA) had received FDA (Food and Drug Administration) approval for the prevention of RSV-related lower respiratory tract disease in patients aged 60 years and over [113]. mRESVIA demonstrated 83.7% efficacy against RSV (95.88% CI, 66.0–92.2), providing protection against both RSV-A and RSV–B subtypes [114]. The majority of adverse reactions related to the vaccine were mild to moderate and transient in nature [114]. mRESVIA employs the same LNP technology as Moderna’s mRNA-based COVID-19 vaccine to deliver an mRNA sequence encoding a stabilised prefusion F viral glycoprotein [113] and is the first mRNA vaccine to gain FDA approval for the prevention of an infectious disease other than COVID-19.

### Zika

Two vaccine candidates, mRNA-1325 and mRNA-1893, have been evaluated in phase I clinical trials (mRNA-1325; NCT03014089 [55], and mRNA-1893; NCT04064905 [56]). Both vaccines were well tolerated, with no serious adverse events reported [115]. However, mRNA-1893 demonstrated favourable immunogenicity, inducing higher levels of neutralising antibodies along with serum-binding antibodies that persisted for up to 13 months in all dosage groups. mRNA-1893 has advanced to a phase II clinical trial (NCT04917861) [57] where its safety, tolerability, and reactogenicity were evaluated in healthy adults in both endemic and non-endemic regions. The results for this study are not yet available.

### Combination mRNA Vaccines

In addition to vaccines against single pathogens the use of combination vaccines, protecting against multiple pathogens, is being explored. The vaccine candidate mRNA-1653, for the combined prevention of human metapneumovirus (HMPV) and parainfluenza virus type 3 (PIV3) has completed clinical trials (NCT04144348 and NCT03392389) [58, 59], although the results are yet to be made available. Another mRNA vaccine candidate for the combined prevention of RSV and HMPV across multiple age groups was investigated in a further clinical trial (NCT06237296) [60], although, again, the results are not yet available.

The combined prevention of RSV and influenza was explored in a phase I/II clinical trial (NCT05788237) [61], investigating the safety and effects of the combined administration of two interventional vaccine candidates, RSVpreF and modRNA qIRV. The combination of the two vaccines was well tolerated with no major safety concerns, and the majority of the adverse events were mild to moderate. The combination did not appear to significantly increase the incidence or severity of adverse events compared to qIRV when administered alone. In terms of serious adverse events, the 0.5 mL dose was better tolerated compared to the higher 1.0 mL dose, but was associated with a higher incidence of systemic adverse events. Further investigation is likely to be required to determine the optimal dose due to the small sample size and the inconsistencies observed in the safety profiles of the 0.5 mL and 1.0 mL doses.

### Completed Clinical Trials for mRNA Vaccine Candidates That Extend Beyond Viral Infections

Although clinical trials using mRNA vaccine candidates against viral infections make up the majority of completed clinical trials using mRNA vaccine candidates, one clinical trial against a parasitic infection has been reported [62]. This phase I clinical trial (NCT05581641) [62] evaluated an mRNA-based vaccine encoding the *Plasmodium falciparum* circumsporozoite protein (PfCSP) and was evaluated at three dose levels (DLs) to select a safe and tolerable dose. This study was completed in September 2024, with results yet to be made available.

### Ongoing Clinical Trials for mRNA Vaccine Candidates Against Viral Infections

#### Herpesviruses

There are more than 100 known herpesviruses, eight of which routinely infect humans, with enormous implications for public health. In addition to the mRNA vaccine candidates that have completed clinical trials, specifically those against CMV [22–24], further clinical trials using the same mRNA vaccine candidates against CMV and mRNA vaccine candidates against Epstein-Barr virus (EBV) and varicella-zoster virus (VZV) are being conducted in ongoing clinical trials. Phase II (NCT04975893) [75] and Phase III (NCT05085366) [76] clinical trials using mRNA-1647, an mRNA vaccine candidate for protection against CMV, are ongoing. The potential of preventing CMV infections in haematopoietic cell transplantation participants is set to be evaluated in a phase I clinical trial (NCT05683457) [63]. A phase II clinical trial (NCT06133010) [102], designated to evaluate the efficacy of the mRNA-1647 vaccine in liver transplant candidates and recipients was recently withdrawn after the trial was cancelled by the sponsor. Withdrawal took effect before participant enrolment and the reason has not been publicly disclosed.

Two mRNA vaccine candidates, mRNA-1195 (NCT05831111) [77] and mRNA-1189 (NCT05164094) [64], are currently being evaluated in phase I and II clinical trials to determine their potential to protect against EBV infection. Both trials are evaluating safety and reactogenicity, but vaccine candidate mRNA-1195 is being used in healthy adult participants between the ages of 18 and 55, while mRNA-1189 is being investigated in healthy individuals between the ages of 10 and 30. mRNA-1195 includes additional mRNA sequences to mRNA-1189 (not specified) encoding EBV latent antigens, with the aim of eliciting immune responses against both the lytic and latent phases of EBV infection. This could potentially prevent EBV-associated diseases, such as post-transplant lymphoproliferative disorders and multiple sclerosis.

An mRNA vaccine candidate against VZV, modRNA (NCT05703607) [78], is currently in phase II of clinical testing. There are two active clinical trials, assessing the safety, reactogenicity, and immunogenicity of IN001 (NCT06375512) [79] and mRNA-1468 (NCT05701800) [80], which are vaccine candidates against herpes zoster, in phases I and I/II respectively.

A major nucleoside-modified trivalent mRNA-LNP vaccine candidate incorporating one entry molecule (i.e., gD2 glycoprotein) and two immune evasion molecules (i.e., gC2 and gE3 glycoproteins) has demonstrated promising results and is now being evaluated in a phase I clinical trial (NCT05432583) [65] in volunteers with recurrent genital herpes, caused by HSV-2. This type of mRNA vaccine is designed to target three distinct viral antigens, with nucleoside modifications to enhance stability, translation efficacy, and control of undesirable immune responses. LNP formulations primarily comprise cationic ionisable lipids, which provide an acid dissociation constant (pKa) almost equivalent to the pH of the endosome during early mRNA encapsulation [116]. Thus, LNPs aid not only in protecting the mRNA for successful encapsulation but also in promoting the efficient endosomal escape of the mRNA molecule into the cytosol [116].

#### Human Immunodeficiency Virus Type 1

Several other promising mRNA-based vaccine candidates against HIV have entered clinical trials. mRNA-1644 encoding eOD-GT8 60mer, is currently being evaluated in a phase I clinical trial (NCT05414786) [81], while a second version of this vaccine, which encodes Core-g28v2 60mer, is also under investigation (NCT05001373) [82]. Both candidates stimulate the production of broadly neutralising antibodies that target highly conserved regions of HIV-1 Env proteins.

A different approach towards the development of an effective HIV-1 vaccine is also being explored in a phase I clinical trial (NCT05217641) [83] of three HIV trimer mRNA vaccine candidates (BG505 MD39.3, BG505 MD39.3 gp151, BG505 MD39.3 gp151 CD4KO). The initial results showed an acceptable safety profile, with most reactions being mild to moderate in severity and no life-threatening events occurring across the vaccine candidates. Early immunogenicity data suggest that gp151-based formulations, especially at the 250 mcg dose level, induce stronger immune responses against BG505/T332N. Robust responses against MW965.26 were also observed across the different vaccine candidates, with the gp151-based formulations showing favourable immunogenicity.

In a further clinical trial (NCT06557785), [66], the safety and immunogenicity of two mRNA-LNP vaccine candidates (CH505M5 N197D mRNA-gp160 and CH505 TF mRNA-gp160) will be evaluated in healthy adults. This is the first-in-human trial for these two vaccine candidates, which aid in the development of broadly neutralising antibodies by expanding and boosting CH235-like B cell precursors. Three other experimental mRNA vaccine candidates, encoding key HIV envelope glycoprotein-derived constructs (eOD-GT8 60mer, Core-g28v2 60mer, and N332-GT5 gp151), are also set to be explored in a phase I clinical trial (NCT06694753) [97], for the prevention of HIV in healthy adults.

### Other Viral Infections

Other ongoing clinical trials evaluating mRNA vaccines against viral infections include those for influenza, Japanese encephalitis and RSV (alone and in combination with HMPV), as shown in Table 2.

### Ongoing Clinical Trials for mRNA Vaccine Candidates That Extend Beyond Viral Infections

A phase I/II clinical trial (NCT05975099) [94], is evaluating the safety and immunogenicity of two candidate vaccines (mRNA-1982 and mRNA-1975) against Lyme disease, in healthy adult participants. Both vaccines target between one and seven serotypes of the outer surface protein A. mRNA-1982 is a monovalent vaccine covering a serotype specific to the bacteria that cause the majority of cases of Lyme disease in the United States, while mRNA-1975 covers seven serotypes that are more prevalent in Europe. The estimated study completion is March 2026.

In addition, two clinical trials using mRNA-based vaccine candidates, encoding multiple *Mycobacterium tuberculosis* antigens, in BCG-naïve healthy volunteers (NCT05547464) [95], and BCG-vaccinated healthy volunteers (NCT05537038) [96], with the aim of selecting a safe and tolerable dose in a three-dose schedule [96] are ongoing. Both studies are active, with estimated study completion dates in 2026 and 2027, respectively.

## Discussion

This systematic review aimed to improve our understanding of the emerging field of mRNA vaccines by providing a comprehensive overview of the clinical trials using mRNA vaccine candidates against infectious diseases other than COVID-19. This systematic review identified clinical trials using mRNA candidate vaccines against 14 viral, two bacterial and one protozoan pathogen, offering prospects for the prevention and control of infectious diseases where conventional vaccine strategies have failed to provide robust protection. The review revealed a limited focus on infectious diseases that extend beyond viral infections, exposing a significant gap within the current mRNA development pipeline. This is likely due to the more complex life cycles of these pathogen groups and highlights the need for future research to expand the use of mRNA vaccines against such pathogens, which remain major contributors to the disease burden. The use of this methodology provides a clear opportunity to address pressing global health concerns, including antimicrobial-resistant (AMR) bacteria and neglected tropical diseases. In addition to the mRNA candidate vaccines being tested in clinical trials multiple preclinical studies in various animal models are underway, or have concluded, with the expectation that if successful these will progress to clinical trials in humans. In addition to the preventative mRNA vaccinations against infectious diseases reported in this systematic review there are preclinical trials with mRNA vaccines against Crimean-Congo haemorrhagic fever [117], Ebola [118] and toxoplasmosis [119].

Of the 83 clinical trials identified in this review 43 trials have concluded, 21 are active, and a further 12 are recruiting, with the remaining not yet recruiting, enrolling by invitation, or withdrawn. Of the 43 completed clinical trials 26 were phase I trials, eight were phase I/II trials, three were phase II trials, and six were phase III trials. One vaccine candidate has been licensed for use in the United States, specifically for the prevention of RSV. One further candidate vaccine, Moderna’s mRNA-1010, designed against influenza, has completed all phases, although a regulatory approval application has yet to be submitted.

This systematic review has a number of limitations. Only clinical trials registered on ClinicalTrials.gov and PubMed were included, and clinical trials were recorded as reported in these databases, such that accuracy is dependent on databases being kept up to date. Discrepancies have been reported between ClinicalTrials.gov recruitment status and actual trial status, as a result of delays in updating the status of said trials on ClinicalTrials.gov [120]. Findings on safety, efficacy, and immunogenicity were reported in this systematic review but as many of the clinical trials evaluated are still active, the results have not yet been made available. The work was limited to preliminary data due to the lack of long-term safety, efficacy, and immunogenicity assessments. Considering the extensive research on mRNA formulations and the considerable progress in the development of mRNA vaccines as preventive and therapeutic tools, additional vaccine candidates are expected to enter clinical trials in the near future, necessitating further updates. Moreover, the variations in mRNA vaccines in terms of formulation, mode of administration, dosage regimens, and target diseases, mean that reaching uniform conclusions can be complex.

Despite these limitations, the results suggest that these mRNA vaccine candidates have good safety profiles and are immunogenic in various experimental studies. In particular, the findings of this systematic review show that some of these mRNA vaccines can stimulate both humoral and cellular immune responses. The advantages of mRNA vaccines over current vaccine technologies include the speed with which they can be designed and manufactured, thereby providing protection from current circulating pathogenic variants. However, many challenges remain around mRNA vaccines that require resolution. The safety, efficacy, and immunogenicity of mRNA vaccines depend heavily on the dosage regimen, mode of administration, and vaccine formulation. Thus, further improvements and modifications to these factors could benefit the overall performance of these vaccines. Therefore, although some mRNA vaccine candidates face limitations, such as stability during storage, suboptimal immunogenicity of certain components, and the lack of appropriate animal models to test certain vaccines, these issues will very likely be addressed through further research and improvements in mRNA technology.

Further research in biologically relevant models and high-risk populations is crucial to ensure high-quality performance within the targeted population. In addition, ensuring the accessibility of mRNA vaccines in endemic and low-income areas by providing cost-effectiveness and stability even at non-ultra-cold storage temperatures is paramount. Given that only one of these vaccines has been licensed for use in humans, and this vaccine was licensed in 2024, their real world efficacy remains to be seen. Long-term safety, efficacy, and immunogenicity studies will further benefit the development of safe and effective mRNA vaccines.

In addition to the proven efficacy of this technology against infectious disease, which was first highlighted during the COVID-19 pandemic, there is considerable interest in the use of mRNA vaccines beyond the prevention of infectious diseases, with several studies showing promise in the use of this technology to treat cancer, including breast cancer [121], melanoma [122], and non-small cell lung cancer [123]. There are also preclinical studies investigating the use of mRNA vaccines against chronic inflammatory conditions including in the murine model of multiple sclerosis [124].

Overall, this systematic review provides an overview of clinical trials using mRNA vaccine candidates against infectious diseases other than COVID-19, and it provides evidence to support the idea that mRNA vaccine technology will shape the future of preventive medicine and have a significant impact on global public health.
